# Prevalence and Risk of Violence and the Physical, Mental, and Sexual Health Problems Associated with Human Trafficking: Systematic Review

**DOI:** 10.1371/journal.pmed.1001224

**Published:** 2012-05-29

**Authors:** Siân Oram, Heidi Stöckl, Joanna Busza, Louise M. Howard, Cathy Zimmerman

**Affiliations:** 1 Section for Women's Mental Health, Institute of Psychiatry, King's College London, London, United Kingdom; 2 Department for Global Health and Development, London School of Hygiene & Tropical Medicine, London, United Kingdom; 3 Department for Population Studies, London School of Hygiene & Tropical Medicine, London, United Kingdom; Medical Research Council, South Africa

## Abstract

**Background:**

There is very limited evidence on the health consequences of human trafficking. This systematic review reports on studies investigating the prevalence and risk of violence while trafficked and the prevalence and risk of physical, mental, and sexual health problems, including HIV, among trafficked people.

**Methods and Findings:**

We conducted a systematic review comprising a search of Medline, PubMed, PsycINFO, EMBASE, and Web of Science, hand searches of reference lists of included articles, citation tracking, and expert recommendations. We included peer-reviewed papers reporting on the prevalence or risk of violence while trafficked and/or on the prevalence or risk of any measure of physical, mental, or sexual health among trafficked people. Two reviewers independently screened papers for eligibility and appraised the quality of included studies. The search identified 19 eligible studies, all of which reported on trafficked women and girls only and focused primarily on trafficking for sexual exploitation. The review suggests a high prevalence of violence and of mental distress among women and girls trafficked for sexual exploitation. The random effects pooled prevalence of diagnosed HIV was 31.9% (95% CI 21.3%–42.4%) in studies of women accessing post-trafficking support in India and Nepal, but the estimate was associated with high heterogeneity (*I* ^2^ = 83.7%). Infection prevalence may be related as much to prevalence rates in women's areas of origin or exploitation as to the characteristics of their experience. Findings are limited by the methodological weaknesses of primary studies and their poor comparability and generalisability.

**Conclusions:**

Although limited, existing evidence suggests that trafficking for sexual exploitation is associated with violence and a range of serious health problems. Further research is needed on the health of trafficked men, individuals trafficked for other forms of exploitation, and effective health intervention approaches.

*Please see later in the article for the Editors' Summary*

## Abstract 2

Siân Oram and colleagues conduct a systematic review of the evidence on the health consequences of human trafficking. They describe a limited and poor-quality evidence base, but some evidence suggests a high prevalence of violence and mental distress among women and girls trafficked for sexual exploitation, among other findings.

## Editors' Summary

**Background:**

The United Nations defines human trafficking as the recruitment and movement of individuals—most often by force, coercion or deception—for the purpose of exploitation. Essentially, human trafficking is the modern version of the slave trade and is a gross violation of human rights. People who have been trafficked may be sold on to the sex industry or forced to work in many forms of labor, including in domestic service and in the agricultural and construction industries. Given the nature of human trafficking, quantifying the scale of the problem is fraught with difficulties, but 2005 statistics estimate that 2.5 million people were in forced labor as a result of being trafficked.

**Why Was This Study Done?:**

To date, the health consequences and public health implications of human trafficking have received little international attention, partly because not much is known about this area. So in this study, the researchers examined published studies in order to assimilate evidence and information on the prevalence of all forms of violence relating to people who have been trafficked and the prevalence of physical, mental, and sexual health problems, including HIV/AIDS, among this group.

**What Did the Researchers Do and Find?:**

The researchers searched the published literature for suitable studies by conducting a comprehensive key word search of key databases and by contacting experts. The researchers did not exclude any type of study from their search but used stringent criteria to identify appropriate studies and then assessed the quality of identified studies by using a critical appraisal tool.

Using this process, the researchers initially identified 407 papers but only 19 were suitable for their analysis, representing 16 different studies. The majority (11) of these studies were conducted in Asia (Nepal, India, Thailand, and Cambodia), and all studies focused solely on women and girls, with all but two studies examining sexual exploitation only.

In their analysis of these studies, the researchers found that women and girls who had been trafficked for sexual exploitation were consistently reported to have experienced high levels of physical and sexual violence. Studies also reported a high prevalence of physical, mental, and sexual health problems among women who had been trafficked and headache, back pain, stomach pain, and memory problems were commonly reported physical health symptoms. The studies that used screening tools to identify mental distress found high levels of anxiety (48.0%–97.7%), depression (54.9%–100%), and post-traumatic stress disorder (19.5%–77.0%). Furthermore, the three studies that examined the associations between trafficking and health suggest that a longer duration of trafficking may be linked to higher levels of mental distress and increased risk of HIV infection. The few studies that examined the prevalence of HIV infection (in women accessing post-trafficking services in India and Nepal) showed a combined prevalence of 31.9%.

**What Do These Findings Mean?:**

These findings, although limited, show that trafficking for sexual exploitation is associated with violence and a range of serious health problems. However, the key finding of this study is that evidence on trafficked people's experiences of violence and of physical, mental, and sexual health problems is extremely limited. There is an enormous gap in research on the health of trafficked men, trafficked children, and people who have been trafficked for labor exploitation. There is an urgent need for more and better information on the needs and experiences of people who have been trafficked, including evidence on effective interventions to mitigate the associated physical and psychological damage.

**Additional Information:**

Please access these Web sites via the online version of this summary at http://dx.doi.org/10.1371/journal.pmed.1001224.

- Humantrafficking.org is a web resource for combatting human trafficking, available in a number of languages
- Stop the Traffick is an international movement campaigning to stop human trafficking, available in a number of languages
- The not for sale campaign works to abolish this form of slavery

## Introduction

Human trafficking is the recruitment and movement of individuals—most often by force, coercion, or deception—for the purpose of exploitation [1]. It is a human rights violation that is criminalized in a growing number of countries [2],[3]. Statistics on the scale of the problem are unreliable, but the International Labour Organisation estimates that globally 2.5 million persons are in situations of forced labour as a result of trafficking [4].

Reports from around the world include descriptions of the extreme forms of physical, psychological, and sexual abuse perpetrated against people who are trafficked for exploitation in the sex industry and a multitude of labour settings, including construction, agriculture, and domestic servitude [5]–[9]. Yet, the health consequences and potential public health implications of human trafficking have generally received little attention. The United Nations Optional Protocol to Prevent, Suppress and Punish Trafficking in Persons, Especially Women and Children [1]—the main international instrument addressing human trafficking—requires state parties to consider implementing measures to promote the recovery of health and, to a limited degree, medical responses to trafficked people's health needs [10]. This systematic review aimed to estimate: (1) The prevalence of violence whilst trafficked and the risk of violence among trafficked people; (2) The prevalence of physical, mental, and sexual health problems, including HIV/AIDS, among trafficked people; and the risk of these problems among trafficked people; (3) The pooled prevalence of violence and of physical, mental, and sexual health problems, including HIV/AIDS, among trafficked people; and the pooled risk of these problems among trafficked people.

## Methods

### Ethics Statement

Ethical approval was not required for this work.

### Selection Criteria

The systematic review protocol is available in Text S1. The review follows PRISMA reporting guidelines (for the PRISMA checklist, see Text S2) [11]. Studies were eligible for inclusion if they: (1) included participants (males or females, adults, or children) who self-identified, or were defined by researchers, as having been trafficked; (2) presented the results of peer-reviewed research based on either a cross-sectional survey; case control study; cohort study; case series analysis; experimental study with baseline measures for the outcomes of interest; or secondary analysis of organisational records; (3) measured either the prevalence of participants' experiences of physical, psychological, or sexual violence while trafficked and/or the prevalence of any reported measure of physical, mental, sexual, and/or reproductive health. No restrictions were placed on study setting. Studies were excluded if they: (1) used a qualitative or single case study design; (2) were published in a book, report, or other non-peer reviewed publication. There were no language restrictions on inclusion for the systematic review.

### Search Strategy

Medline, PubMed, EMBASE, Web of Science, and PsycINFO were searched from date of inception to 31st August 2011 using a combination of Medical Subject Headings (MeSH) and text words. The search strategy is shown in Text S3. These electronic searches were supplemented by screening the reference lists of include papers, citation tracking, and expert recommendations. Replies were received from 14 of 17 experts contacted. Due to time and resource constraints, hand searches of journals were not carried out.

### Data Extraction

Two reviewers (SO and HS) screened the downloaded titles and abstracts against the inclusion criteria. When it was unclear whether a citation was relevant, it was included for retrieval. Two reviewers (SO and HS) then assessed the full text of potentially eligible papers against the inclusion criteria. If it was considered that studies had collected relevant data but had not presented it (e.g., if trafficked people were included in the study sample but outcomes data were not presented according to trafficking status), we contacted authors to request further information. Data from the included papers were extracted by SO; HS independently extracted data from a random sample of 20% of papers as a check; no differences were found. Data were extracted on study design; sample characteristics; the definition and method of assessing human trafficking; measurement of experiences of violence and health problems; and outcomes. Where possible, outcome measures were extracted separately by gender, age, and type of exploitation.

### Quality Appraisal

Using criteria adapted from the Critical Appraisal Skills Programme (CASP), two reviewers (SO and HS) independently appraised the quality of included studies [12]. The quality appraisal form (see Text S4) includes 15 questions about study quality; papers receive a grade of between 0 and 2 for each question, giving a maximum total score of 30. The reviewers compared their quality appraisal scores and resolved any disagreements before calculating the final appraisal score. Sub-scores for questions relating to the quality of studies' sampling and measurement strategies were also calculated. Assessment of sampling quality focused on sampling methods, sample characteristics, and the participation rate. Assessment of measurement quality focused on the measurement of the exposure (having been trafficked), outcomes (violence and/or physical, mental or sexual health problems), and known or potential confounders. Quality scores are reported in Table 1.

**Table 1:**
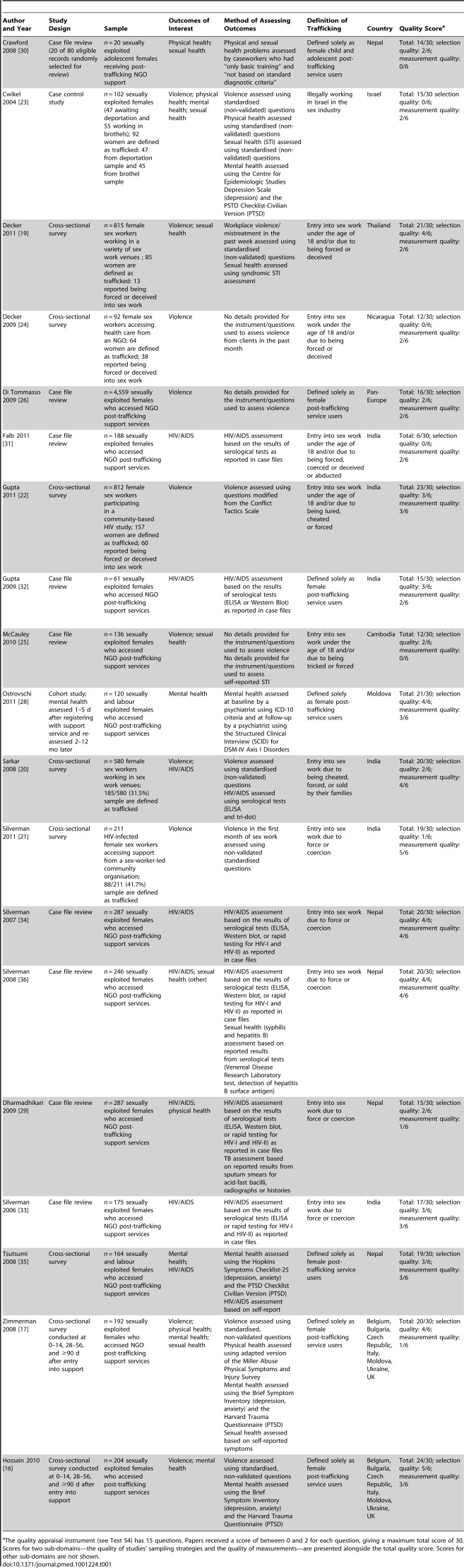
Characteristics of included papers (*n* = 19).

a The quality appraisal instrument (see Text S4) has 15 questions. Papers received a score of between 0 and 2 for each question, giving a maximum total score of 30. Scores for two sub-domains—the quality of studies' sampling strategies and the quality of measurements—are presented alongside the total quality score. Scores for other sub-domains are not shown.

### Data Analysis

Information about the study design, study sample, definition and method of assessing human trafficking, and method of assessing violence and health problems was summarised. Prevalence estimates and odds ratios (ORs) for violence and/or health outcomes were calculated separately by gender, age, and type of exploitation when appropriate data were provided.

Meta-analysis of the prevalence of HIV infection among trafficked women was carried out using a random effects model, generating a pooled prevalence with 95% CIs. Heterogeneity among studies was estimated using the *I* ^2^ statistic, which describes the percentage of variation across studies that is due to heterogeneity rather than chance [13]. Analyses were conducted in STATA 11.2 using the *metan* command [14]. Pooled estimates were not, however, calculated for other outcomes because of limited use of diagnostic tests and poor study comparability.

### Bias

Where the review identified multiple eligible papers from the same study, only the most definitive results were included for each outcome of interest. Restricting the review to peer-reviewed publications resulted in the exclusion of two reports on the health of women trafficked for sexual exploitation [5],[15]. Two of the papers included in this review were, however, drawn from the study on which these reports are based [16],[17]. A report from Eastern Africa that included the health of trafficked people but that was not peer-reviewed was also excluded [18].

## Results

**Figure 1:**
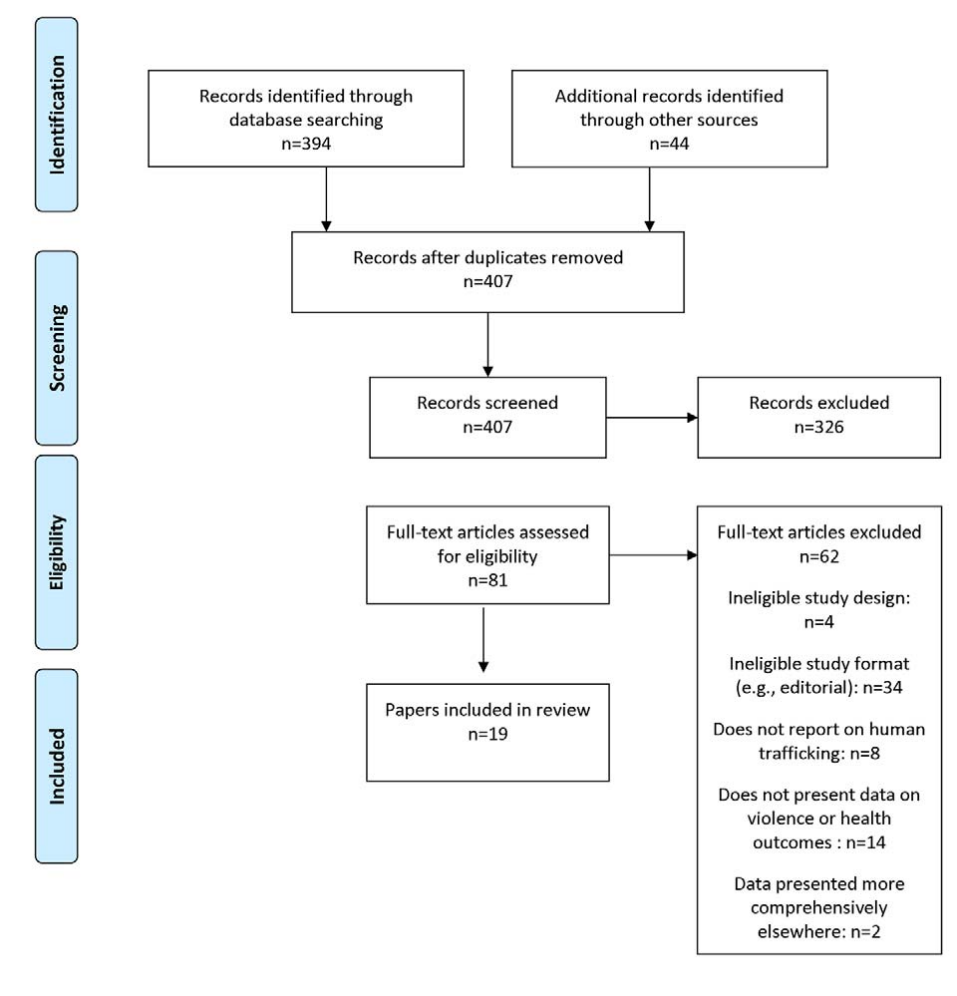
Flowchart of primary study selection.

The study selection process is presented in Figure 1. Our literature searches returned 407 unique records, of which 326 were excluded following title and abstract screening. Full text copies of the remaining 81 references that met, or potentially met, the inclusion criteria were retrieved. After further screening, 19 papers were retained for inclusion. Of the 19 included papers, 18 were identified from searches of electronic databases, 0 from citation tracking, and one from expert recommendations. None of the included papers were published in a language other than English.

### Key Features of Included Papers

Table 1 summarises the key features of the 19 included papers. Papers that report on the same studies are grouped together. The 19 papers reported on 16 studies, 11 of which were conducted in South or Southeast Asia (Nepal, India, Thailand, and Cambodia), four in Europe, and one in Central America. All of the studies were conducted with trafficked women or girls and the majority (14/16) focused on sexual exploitation only. Ten of the 16 studies were conducted with women and girls who were accessing post-trafficking support services. The remaining six studies were conducted in alternative settings and their samples included non-trafficked women. Nine studies collected data on violence, four on physical health, four on mental health, six on HIV/AIDS, and six on other aspects of sexual health.

### Violence

Six studies compared the prevalence of violence reported by women identified as trafficked into sex work and non-trafficked sex workers (Table 2). Three studies collected data on the prevalence of violence at or shortly after women's entry into sex work and reported that the odds of violence were significantly higher for trafficked women versus non-trafficked sex workers [19]–[21]. Estimates of the prevalence and risk of violence in other time periods were more variable: two studies conducted in India and Thailand reported that the odds of workplace violence were significantly higher for trafficked women than for non-trafficked sex workers [19],[22], whereas two studies conducted in Nicaragua and Israel detected no difference between the two groups [23],[24]

**Table 2:** Prevalence and risk of violence whilst trafficked (*n* = 9).

Author and Year	Type of Violence	Frequency of Violence (Trafficked People)	Frequency of Violence (Controls)	OR and 95% CI
**Sex industry samples**				
Cwikel 2004 [23]	Physical assault at work	30/93 (32.3%)	2/10 (20.0%)	1.9 (0.35–19.4)
	Sexual assault at work	20/93 (31.2%)	1/10 (10.0%)	2.5 (0.31–113.4)
Decker 2011 [19]	Physical, sexual or psychological violence or mistreatment at work in the past week	44/85 (51.8%)	254/730 (34.8%)	2.0 (1.25–3.24)
	Sexual violence at initiation into sex work	10/85 (11.8%)	26/730 (3.6%)	3.6 (1.49–8.09)
Decker 2009 [24]	Physical or sexual violence from a client in the past month.			
	(1) Entry age <18 or forced or deceived into sex work	31/62 (50.0%)	10/28 (35.7%)	1.8 (0.66–5.08)
	(2) Entry <18 y	20/38 (52.6%)	10/28 (35.7%)	2.0 (0.66–6.18)
	(3) Forced or deceived into sex work	17/37 (45.9%)	10/28 (35.7%)	1.53 (0.50–4.76)
Gupta 2011 [22]	Any violence in the past 6 mo			
	(1) Entry age <18 or forced or deceived into sex work	84/157 (53.5%)	256/655 (39.1%)	1.79 (1.26–2.54)
	(2) Entry <18 y, not forced or deceived into sex work	50/96 (52.1%)	256/655 (39.1%)	1.69 (1.08–2.67)
	(3) Entry age <18 y and forced or deceived into sex work	16/26 (61.5%)	256/655 (39.1%)	2.49 (1.04–6.24)
	(4) Entry >18 y and forced or deceived into sex work	18/34 (52.9%)	256/655 (39.1%)	1.75 (0.83–3.74)
Sarkar 2008 [20]	Physical, sexual, or psychological violence in the first few months after entry into sex work	105/183 (57.3%)	61/397 (15.3%)	7.4 (4.8–11.3)
Silverman 2011 [21]	Any violence in the first month after entry into sex work	66/88 (75.0%)	66/123 (53.7%)	2.6 (1.4–4.7)
**Post-trafficking support service samples**				
Di Tommaso 2009 [26]	Any violence or material neglect while trafficked	1,350/1,644 (82.1%)	—	—
McCauley 2010 [25]	Physical violence while trafficked	13/136 (9.6%)	—	—
	Sexually abused while trafficked	42/136 (30.9%)	—	—
Zimmerman 2008 [17]	Physical or sexual violence while trafficked	182/192(94.8%)	—	—
	Physical violence while trafficked	145/192 (75.5%)	—	—
	Sexual violence while trafficked	172/192 (89.6%)	—	—

Several studies categorised women as trafficked if they reported either having been lured, tricked, or cheated into sex work or that they had begun sex work under the age of 18 [22],[24],[25]. Decker et al. provided supplementary data for the review that indicated that, in their survey of Nicaraguan female sex workers, 52.6% of women who reported entering sex work under the age of 18 reported past month victimisation by a client compared with 45.9% of women who were forced or coerced into sex work [24]. Gupta et al. reported that the highest odds of violence were among women who had been forced or deceived into entering sex work under the age of 18 [22].

Three studies that were conducted with trafficked women who were accessing post-trafficking support services from non-governmental organisations (NGOs) reported on violence [17],[25],[26]. Zimmerman et al.'s estimate of a 94.8% prevalence of violence among trafficked women [17] compares with the highest recorded national rates of gender-based violence in the world [27]. A comparatively low level of physical violence was reported by McCauley et al.: their review of case files from 26 Cambodian NGOs found that 9.6% of women and girls reported physical violence and 33.1% reported sexual violence [25].

### Physical Health

Three studies collected data on trafficked women's physical health symptoms [17],[23],[29]. Zimmerman et al. reported that, when interviewed between 0 and 14 d after entering into post-trafficking support services, 63% of participants reported suffering from ten or more concurrent symptoms [17]. Among the most common physical health problems reported were headaches (82.3%), fatigue (81.3%), dizziness (70.3%), back pain (68.8%), and memory problems (62.0%) [17]. Commonly reported symptoms among Cwikel's brothel and detention-based samples similarly included headache, dizziness, back and stomach pain, and dental problems [23].

Among adolescents, Crawford and Kaufman's case file review also identified headaches (35%), stomach pains (25%), pelvic pain (15%), skin conditions (10%), and fatigue (10%) [30]. The authors note, however, that the case files contained minimal detail and that “diagnoses” were made by NGO counsellors with only basic training. It is also unclear after how much time following entry into care services these symptoms were reported.

### Sexual Health

**Figure 2:**
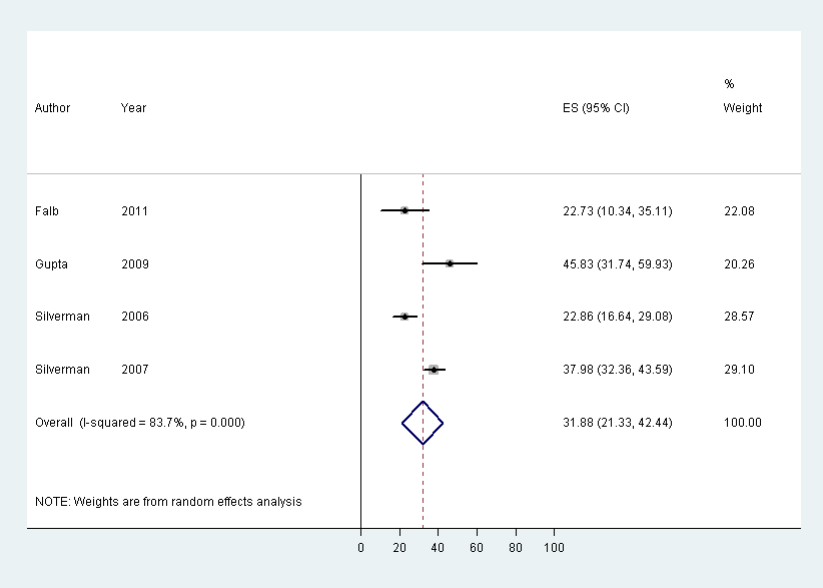
Pooled prevalence of HIV infection among trafficked women receiving post-trafficking support services in India and Nepal.

Data on the prevalence of HIV infection among trafficked women were available only from studies conducted in India and Nepal. Four studies, reporting data from the serological test results recorded in the case files of women receiving post-trafficking support services, estimated that the prevalence of HIV ranged from 22.7% to 45.8% (Table 3) [31]–[34]. The random effects pooled prevalence for these four studies was 31.9% (95% CI 21.3%–42.4%) (Figure 2). This estimate was associated with high heterogeneity (*I* ^2^ = 83.7%). A lower prevalence of infection was reported by Sarkar et al. in a survey of trafficked (13.1%) and non-trafficked sex workers (10.1%), which also reported that the odds of infection did not differ significantly between the two groups (Table 3) [20]. Tsutsumi et al.'s report of a zero prevalence of HIV infection among women trafficked for labour exploitation should be treated with caution: infection status was self-reported and 80.0% of women trafficked for labour exploitation reported that they did not know their HIV status [33].

**Table 3:** Prevalence and risk of HIV infection among trafficked women (*n* = 6).

Author and Year	Method Used to Assess HIV	Frequency of HIV Infection (Trafficked People)	Frequency of HIV Infection (Controls)	OR and 95% CI
Sex industry samples				
Sarkar 2008 [20]	Serological tests (ELISA and tri-dot)	24/183 (13.1%)	40/397 (10.1%)	1.35 (0.75–2.4)
**Post-trafficking support service samples**				
Falb 2011 [31]	Test results as recorded in case files	10/44 (22.7%)	—	—
Gupta 2009 [32]	Serological test results as recorded in case files (ELISA or Western Blot)	22/48 (45.8%)	—	—
Silverman 2006 [33]	Serological test results as recorded in case files (ELISA or rapid testing for HIV-I and HIV-II)	40/175 (22.9%)	—	—
Silverman 2007 [34]	Serological test results as recorded in case files (ELISA, Western blot, or rapid testing for HIV-I and HIV-II).	109/287 (38.0%)	—	—
Tsutsumi 2008 [35]	Self-report	Sexual exploitation 13/44 (29.6%)	—	—
		Labour exploitation 0/120 (0.0%)	—	—

Silverman et al. investigated the risk factors associated with HIV infection among trafficked women receiving post-trafficking support in India and in Nepal [33],[34]. In the Nepalese study, the odds of infection were significantly increased among women who had been trafficked at age 14 or younger when compared to women who had been trafficked at age 18 or older (OR 3.42, 95% CI 1.51–7.75); had been trafficked to Mumbai versus other cities (OR 6.27, 95% CI 3.04–12.9); and had been forced into prostitution for a greater number of months (OR 1.02, 95% CI 1.01–1.04) [20]. In the Indian study, longer duration of exploitation was also associated with higher odds of HIV infection (OR 1.04, 95% CI 1.02–1.06), but younger age when initially trafficked was not. Instead, the risk of HIV infection was found to vary significantly with women's place of origin [33].

Only one study reported on the results of serological tests for sexually transmitted infections: Silverman et al. reported that among women accessing post-trafficking support in Nepal, the prevalence of infection with syphilis and hepatitis B was 0.4% and 3.8%, respectively [36]. A further five studies reported on the prevalence of self-reported symptoms of sexually transmitted or other gynaecological infections among trafficked women, which ranged from 5.7% to 65.9% [17],[19],[23],[25],[30]. Participants' self-reported symptoms, however, may not be a reliable indicator of infection [37],[38]. Two of these studies compared the prevalence of self-reported symptoms of sexually transmitted and other gynaecological infections in trafficked and non-trafficked sex workers; neither reported a significant difference between the two groups [19],[23].

### Mental Health

Only one study used a validated diagnostic instrument to assess psychiatric disorder among trafficked women (Table 4): Ostrovschi et al. used the Structured Clinical Interview for DSM disorders to diagnose disorder among trafficked women 2–12 mo after they had returned to Moldova and registered for post-trafficking support services [28],[39]. 16.7% of women were diagnosed with depression and 35.8% were diagnosed with post-traumatic stress disorder (PTSD). Three other studies screened women for, but did not attempt to diagnose, mental disorder and the prevalence of anxiety, depression, and PTSD they reported varied considerably (Table 4) [16],[23],[25].

**Table 4:** Prevalence and risk of mental distress among trafficked women (*n* = 4).

Author and Year	Instrument and Threshold Used to Assess Mental Distress	Frequency of Mental Distress (Trafficked People)	Frequency of Mental Distress (Controls)	OR and 95% CI
**Anxiety**				
Hossain 2010 [16]	Brief Symptom Inventory Mean score ≥1.87	98/204 (48.0%)	-	—
Tsutsumi 2008 [35]	Hopkins Symptoms Checklist 25 score ≥1.75	Sexual exploitation 43/44 (97·7%)	—	—
		Labour exploitation 105/120 (87·5%)	—	—
**Depression**				
Cwikel 2004 [23]	Centre for Epidemiologic Studies Depression Scale mean score	48/84 (57.1%)	2/7 (28.6%)	3.33 (0.50–36.41)
Hossain 2010 [16]	Brief Symptom Inventory mean score ≥1.87	112/204 (54.9%)	—	—
Ostrovschi 2011 [28]	Clinical interview using ICD-10 criteria (baseline)	4/120 (3.3%)	—	—
	Diagnostic assessment using Structured Clinical Interview for DSM-IV disorders (follow up)	24/120 (16.7%)	—	—
Tsutsumi 2008 [35]	Hopkins Symptoms Checklist 25 score ≥1·75	Sexual exploitation 44/44 (100.0%)	—	—
		Labour exploitation 97/120 (81.8%)	—	—
**Post-traumatic stress disorder**				
Cwikel 2004 [23]	PTSD Checklist Civilian Version score ≥50	17/87 (19.5%)	1/7 (14.3%)	1.46 (0.16–70.90)
Hossain 2010 [16]	Harvard Trauma Questionnaire, mean score ≥2.00	157/204 (77.0%)	—	—
Ostrovschi 2011 [28]	Clinical interview using ICD-10 criteria (baseline)	58/120 (48.3%)	—	—
	Clinical interview using Structured Clinical Interview for DSM disorders (follow up)	43/120 (35.8%)	—	—
Tsutsumi 2008 [35]	PTSD Checklist Civilian Version score ≥50	Sexual exploitation 13/44 (29.5%)	—	—
		Labour exploitation 9/120 (7.5%)	—	—

ICD, International Classification of Diseases.

Hossain et al. reported that violence and injuries sustained whilst trafficked were associated with an increased risk of high levels of symptoms of anxiety, depression, and PTSD [16]. After adjusting for exposure to violence prior to and during exploitation, women who had been exploited for 6 mo or more reported significantly higher symptom levels of depression (adjusted OR [AOR] 2.23, 95% CI 1.11–4.53) and anxiety (AOR 2.22, 95% CI 1.11–4.46) [16]. After the same adjustments, women who had been out of the trafficking situation for ≥3 mo reported significantly lower symptom levels of depression (AOR 0.4, 95% CI 0.20–0.8) and anxiety (AOR 0.39, 95% CI 0.2–0.8).

Supplementary data provided by Cwikel enabled a comparison to be drawn between the risk of mental distress among trafficked and non-trafficked sex workers. This analysis, which suffered from a lack of power, found that a higher proportion of trafficked women than non-trafficked sex workers screened positive for both depression (57.1% versus 28.6%) and PTSD (19.5% versus 14.3%), but that the difference was not significant [23]. Research by Tsutsumi et al. reported a high prevalence of anxiety (87.5%) and depression (81.8%) among women trafficked for labour exploitation. The study also suggested significantly increased risk of depression and PTSD among women who had been trafficked for sexual exploitation compared with women who had been trafficked for labour exploitation [35].

## Discussion

### Key Findings

We found that women and girls who had been trafficked for sexual exploitation were consistently reported to have experienced high levels of physical and sexual violence. Studies also reported a high prevalence of physical, mental, and sexual health problems among the trafficked women in their samples. Headache, back pain, stomach pain, and memory problems were commonly reported physical health symptoms. In one study, depression and PTSD were diagnosed among 16.7% and 35.8% of trafficked women who had returned to their country of origin [28]. Other studies, which used screening instruments to identify mental distress, reported a high prevalence of symptoms indicative of anxiety (48.0%–97.7%), depression (54.9%–100%), and PTSD (19.5%–77.0%). Data on the prevalence of HIV infection were available only from studies conducted in India and Nepal. The random effects pooled prevalence of diagnosed HIV infection among women accessing post-trafficking support services in these countries was 31.9% (95% CI 21.3–42.4%). The high heterogeneity associated with this pooled estimate, however, warrants caution.

Only three studies examined associations between the characteristics of the trafficking experience and trafficked women's health. This limited evidence suggests that a longer duration of trafficking may be linked to higher levels of mental distress, which decreases as time since exiting exploitation increases [16], and that the risk of HIV and sexually transmitted infection (STI) may be related as much to prevalence in women's areas of origin and exploitation as other characteristics of exploitation (such as duration and number of clients) [33],[34]. Further research is required, however, to explore the multiple pathways through which trafficking influences various aspects of health.

Whether there are differences between exposure to violence and the health needs of women who are identified as “trafficked” and those working within the same industries who are not identified as such is an important political and service question. Yet, current evidence does not appear sufficiently robust to permit conclusions to be drawn. The studies included in this review suggest that trafficked women are at increased risk of violence at or shortly after entering into sex work. Evidence on whether trafficked women experience more violence than non-trafficked sex workers over longer periods, however, is unclear. Future research on trafficking-related violence should not be limited to violence that individuals experience in the work place, but should also include violence perpetrated by, for example, traffickers and partners.

The review indicates that not all women who had been trafficked for sexual exploitation report experiencing violence, including sexual violence. This raises two issues for researchers and, indeed, for professionals working with trafficked people within the context of criminal or immigration proceedings and support programmes. First, while many women who have been trafficked for sexual exploitation may report having experienced violence, not all will. The absence of physical and sexual violence should not be to the detriment of a person's claim to have been criminally exploited. Second, it should be recognised that not all women who have been trafficked for sexual exploitation will necessarily define their experiences in the sex industry as sexually violent. As with other research on interpersonal violence, disclosure is likely to be enhanced by the use of survey instruments that ask behaviourally specific, rather than subjective questions about violence and by appropriate fieldworker training. In this review, only one study based questions about violence on a validated survey instrument [22]; the majority of studies used standardised but non-validated questionnaires or single questions to enquire about women's experiences of violence.

### Limitations of the Review

The search strategy did not include hand-searching and excluded non-peer reviewed literature. We also identified a number of methodological and conceptual problems in the primary studies that limit the conclusions that can be drawn from the review. Following quality appraisal by two reviewers, nine of the 18 included papers were judged to score <3/6 on questions relating to selection bias. We found that most studies used non-probability sampling and did not provide information on the representativeness of their samples. The generalisability of findings from studies that recruited trafficked people from post-trafficking support services is likely to be particularly limited, as most trafficked persons probably do not access support. If trafficked persons who receive NGO support represent more extreme cases of abuse, studies may overestimate the health risks and consequences of trafficking. Conversely, if they represent individuals who are less damaged and more able to contact and use services, findings may underestimate risk. Similarly, studies based on sex worker samples may under-sample women who are currently trafficked and, as women in particularly confined circumstances are unlikely to be able to participate in research, may primarily—or only—reach those in less restrictive trafficking situations. In this review it was not possible to assess the direction or impact of potential selection bias because we cannot know either the ratio of detected to undetected cases or whether there are systematic differences between the experiences of people who do and do not access care.

The reliability and comparability of primary studies were also limited by the methods of data collection and instruments used to assess trafficked women's experiences of violence and of health problems. Nine of the 18 included papers reported on the results of case file reviews, which are likely to underestimate the prevalence of violence and physical, mental, and sexual health problems. All but one study relied on diagnostic test results when reporting the prevalence of HIV infection in their samples, but in five out of six studies the prevalence of other sexually transmitted or gynaecological infections was based on participants' self-reported symptoms. Only one study used a validated diagnostic instrument to assess mental disorder among trafficked women [28]. No studies used unmodified validated instrument to assess women's physical health symptoms or their experiences of violence while trafficked. The validation of instruments to assess violence and health problems among populations of trafficked people is needed to support the conduct of more rigorous and comparable studies in the future.

Finally, heterogeneity in studies' definitions of “human trafficking” is likely to further reduce the comparability of findings. The definitional complexities of human trafficking have regularly complicated attempts to study the issue. Definitions of human trafficking, and researchers' interpretation of the definition negotiated in 2000 as part of the UN Optional Protocol to Prevent, Suppress and Punish Trafficking in Persons, Especially Women and Children [1], varied substantially. Several studies, for example, categorised any women who reported having been younger than 18 y when they were first paid for sex as having been trafficked [19],[22],[24],[25],[31]. This definition, however, risks the conflation of human trafficking and child prostitution. A further study categorised women as trafficked or non-trafficked on the basis of their immigration status and not on their experiences of, for example, forced or coercive recruitment into the sex industry [23]. A number of studies avoided definitional problems by studying women and adolescents who were using post-trafficking support services [16],[17],[26],[28],[35]. Yet, these studies may have limited comparability if the definitions of who was eligible for support varied between post-trafficking services.

### Future Research Priorities

The limited number of studies identified for inclusion in this review highlights that human trafficking is an emerging field of study whose methodological approaches are in their nascent stages. All of the included studies reported on the experiences of trafficked women and girls and only two included people who had been trafficked for labour exploitation. Tsutsumi and colleagues' finding that 80.0% of the women in their sample who had been trafficked for labour exploitation did not know their HIV status may further reflect the relative attention given to the clinical assessment and care needs of individuals trafficked for labour exploitation. There is, therefore, an enormous gap in research on the health of trafficked men, trafficked children, and people who have been trafficked for labour exploitation. Further research is also needed to identify the similarities and differences between the health risks and problems experienced by trafficked and non-trafficked workers in different industries, including the sex industry, in order to differentiate between harm that may be related directly to trafficking experiences versus harm associated with the particular form of exploitation for which a person has been trafficked. Such research would also suggest how existing expertise about, for example, working with non-trafficked sex workers, could be applied to meeting the health needs of trafficked people. Research is also needed on mechanisms coping and resilience among trafficked people and on how health problems impact functioning. Finally, longitudinal research and intervention trials are required to explore potential strategies to improve the physical and mental health of trafficked persons and foster holistic recovery. As set out in the “WHO Ethical and Safety Recommendations for Interviewing Trafficked Women” [40], research with trafficked people must prioritise the safety and psychological well-being of participants.

### Conclusions

Despite a dramatic increase in the profile of human trafficking over the past decade, the evidence on trafficked people's experiences of violence and of physical, mental, and sexual health problems is extremely limited. Nevertheless, findings from studies to date indicate that trafficking is associated with serious health problems and suggest that trafficked people are likely to require a coordinated response by health care providers and other support services. As there is no sign that human trafficking is abating, we need more and better information on trafficked people's health needs and experiences, including evidence on interventions to mitigate the physical and psychological damage associated with this global crime.

## Supporting Information

Text S1 Protocol for “Prevalence and risk of violence and the physical, mental and sexual health problems associated with human trafficking: systematic review.”
(DOC)

Text S2 PRISMA checklist.
(DOC)

Text S3 Search terms used for Ovid Medline, EMBASE, and PSYCInfo.
(DOC)

Text S4 Quality appraisal checklist.
(DOC)

## Abbreviations

AOR: adjusted odds ratio
NGO: non-governmental organisation
OR: odds ratio
PTSD: post-traumatic stress disorder
STI: sexually transmitted infection

## References

[1] United Nations (2000). Optional Protocol to Prevent, Suppress and Punish Trafficking in Persons, Especially Women and Children, Supplementing the United Nations Convention Against Transnational Organized Crime, G.A. Res. 55/25 (2000).

[2] Council of Europe (2005). Council of Europe Convention on Action against Trafficking in Human Beings and its Explanatory Report.

[3] UNHCR (2002). Principles and guidelines on human rights and trafficking.

[4] ILO (2005). A Global Alliance Against Forced Labour.

[5] Zimmerman C, Hossain M, Yun K (2006). Stolen smiles: The physical and psychological health consequences of women and adolescents trafficked in Europe.

[6] Surtees R (2008). Trafficking of men - a trend less considered: the case of Belarus and Ukraine.

[7] Animus Association (2001). Trafficking in women: questions and answers.

[8] ASI (2006). Trafficking for Forced Labour in Europe.

[9] ASI (2006). Trafficking in women, forced labour and domestic work in the context of the Middle East and Gulf Region.

[10] United Nations (2000). Optional Protocol to Prevent, Suppress and Punish Trafficking in Persons, Especially Women and Children, Supplementing the United Nations Convention Against Transnational Organized Crime (art 6), G.A. Res. 55/25 (2000).

[11] Moher D, Liberati A, Tetzlaff J, Altman DG, The PRISMA Group (2009). Preferred Reporting Items for Systematic Reviews and Meta-Analyses: The PRISMA Statement.. PLoS Med.

[12] Public Health Resource Unit (2006). CASP appraisal tools.. http://www.sph.nhs.uk/sph-files/casp-appraisal-tools.

[13] Higgins J, Thompson S (2002). Quantifying heterogeneity in a meta-analysis.. Stat Med.

[14] StataCorp (2009). Stata Statistical Software: Release 11.

[15] Zimmerman C, Yun K, Shvab I, Watts C, Trappolin L (2003). The health risks and consequences of trafficking in women and adolescents: findings from a European study. Including: human rights analysis of health and trafficking and principles for promoting the health rights of trafficked women.

[16] Hossain M, Zimmerman C, Abas M, Light M, Watts C (2010). The relationship of trauma to mental disorders among trafficked and sexually exploited girls and women.. Am J Public Health.

[17] Zimmerman C, Hossain M, Yun K, Gajdadziev V, Guzun N (2008). The health of trafficked women: a survey of women entering posttrafficking services in Europe.. Am J Public Health.

[18] IOM (2008). Human Trafficking in Eastern Africa. Research Assessment and Baseline Information in Tanzania, Kenya, Uganda and Burundi.

[19] Decker MR, McCauley HL, Phuengsamran D, Janyam S, Silverman JG (2011). Sex trafficking, sexual risk, sexually transmitted infection and reproductive health among female sex workers in Thailand.. J Epidemiol Community Health.

[20] Sarkar K, Bal B, Mukherjee R, Chakraborty S, Saha S (2008). Sex-trafficking, violence, negotiating skill, and HIV infection in brothel-based sex workers of eastern India, adjoining Nepal, Bhutan, and Bangladesh.. J Health Popul Nutr.

[21] Silverman JG, Raj A, Cheng DM, Decker MR, Coleman S (2011). Sex trafficking and initiation-related violence, alcohol use and HIV risk among HIV+ female sex workers in Mumbai.. J Infect Dis.

[22] Gupta J, Reed E, Kershaw T, Blankenship KM (2011). History of sex trafficking, recent experiences of violence, and HIV vulnerability among female sex workers in coastal Andhra Pradesh, India.. Int J Gynecol Obstet.

[23] Cwikel J, Chudakov B, Paikin M, Agmon K, Belmaker R (2004). Trafficked female sex workers awaiting deportation: Comparison with brothel workers.. Arch Women Ment Hlth.

[24] Decker MR, Mack KP, Barrows JJ, Silverman JG (2009). Sex trafficking, violence victimization, and condom use among prostituted women in Nicaragua.. Int J Gynecol Obstet.

[25] McCauley HL, Decker MR, Silverman JG (2010). Trafficking experiences and violence victimization of sex-trafficked young women in Cambodia.. Int J Gynaecol Obstet.

[26] Di Tommaso ML, Shima I, Strom S, Bettio F (2009). As bad as it gets: Well-being deprivation of sexually exploited trafficked women.. European Journal of Political Economy.

[27] Watts C, Zimmerman C (2002). Violence against women: global scope and magnitude.. Lancet.

[28] Ostrovschi NV, Prince MJ, Zimmerman C, Hotineanu MA, Gorceag LT (2011). Women in post-trafficking services in moldova: diagnostic interviews over two time periods to assess returning women's mental health.. BMC Public Health.

[29] Dharmadhikari AS, Gupta J, Decker MR, Raj A, Silverman JG (2009). Tuberculosis and HIV: a global menace exacerbated via sex trafficking.. Int J Infect Dis.

[30] Crawford M, Kaufman MR (2008). Sex trafficking in Nepal: survivor characteristics and long-term outcomes.. Violence Against Wom.

[31] Falb KL, McCauley HL, Decker MR, Sabarwal S, Gupta J (2011). Trafficking mechanisms and HIV status among sex-trafficking survivors in Calcutta, India.. Int J Gynaecol Obstet.

[32] Gupta J, Raj A, Decker MR, Reed E, Silverman JG (2009). HIV vulnerabilities of sex-trafficked Indian women and girls.. Int J Gynaecol Obstet.

[33] Silverman JG, Decker MR, Gupta J, Maheshwari A, Patel V (2006). HIV prevalence and predictors among rescued sex-trafficked women and girls in Mumbai, India.. JAIDS.

[34] Silverman JG, Decker MR, Gupta J, Maheshwari A, Willis BM (2007). HIV prevalence and predictors of infection in sex-trafficked Nepalese girls and women.. JAMA.

[35] Tsutsumi A, Izutsu T, Poudyal AK, Kato S, Marui E (2008). Mental health of female survivors of human trafficking in Nepal.. Soc Sci Med.

[36] Silverman JG, Decker MR, Gupta J, Dharmadhikari A, Seage IGR (2008). Syphilis and hepatitis B co-infection among HIV-infected, sex-trafficked women and girls, Nepal.. Emerg Infect Dis.

[37] Hawkes S, Morison L, Foster S, Gausia K, Chakraborty J (1999). Reproductive-tract infections in women in low-income, low-prevalence situations: assessment of syndromic management in Matlab, Bangladesh.. Lancet.

[38] Ray K, Muralidhar S, Bala M, Kumari M, Salhan S (2009). Comparative study of syndromic and etiological diagnosis of reproductive tract infections/sexually transmitted infections in women in Delhi.. International J Infect Dis.

[39] First M, Spitzer R, Gibbon M, Williams J (2002). Structured Clinical Interview for DSM-IV-TR Axis I Disorders, Research Version, Non-patient Edition (SCID-I/NP).

[40] Zimmerman C, Watts C (2003). WHO Ethical and Safety Recommendations for Interviewing Trafficked Women.

