# Supplementary material for: Prevalence and Risk of Violence and the Physical, Mental, and Sexual Health Problems Associated with Human Trafficking: Systematic Review
Source: PLoS Med. 2012 May 29;9(5):e1001224. doi: 10.1371/journal.pmed.1001224 (PMC3362635; doi:10.1371/journal.pmed.1001224)
Supplement: Text S1 — Protocol for “Prevalence and risk of violence and the physical, mental and sexual health problems associated with human trafficking: systematic review.” (DOC) [file pmed.1001224.s001.doc]

**PROTOCOL: Prevalence and risk of violence and poor physical, mental, and sexual and reproductive health consequences amongst trafficked people; systematic review.**

1. **Title:** Prevalence and risk of violence and poor physical, mental, and sexual and reproductive health consequences amongst trafficked people; systematic review.
2. **Background:**

Human trafficking is a serious crime and a violation of human rights which affects the lives of thousands of people around the world each year. International instruments, such as the United Nations Optional Protocol on the Prevention, Suppression and Punishment of Trafficking in Persons, Especially Women and Children and the Council of Europe Convention on Action against Trafficking in Human Beings recognise the potential for trafficking to result in serious health consequences and the need to meet the health needs of trafficked people . To date, however, there is little guidance available on how to plan, assess or provide for the health needs of trafficked people or awareness of how the health experiences and needs of trafficked people compare to those of non-trafficked people working in the same industry (e.g., the sex industry, and in agriculture, construction and other labour sectors).

A small but growing body of evidence suggests that victims of trafficking for sexual exploitation experience high levels of violence and a range of poor physical, mental and sexual health outcomes as a result of their experiences whilst trafficked. Zimmerman et al, for example, conducted a multi-country study of 192 women trafficked for sexual exploitation in Europe and found that 94.8% women reported experiencing physical or sexual violence and that 63% reported suffering from 10 or more concurrent health symptoms . In particular, women reported suffering from headaches, dizziness, and stomach and back pain. A study by Tsutsumi et al on women trafficked for sexual exploitation and labour exploitation found high levels of symptoms of depression, anxiety and PTSD amongst both groups and high self-reported prevalence of HIV infection amongst sex trafficked women .

The aim of this review is to synthesise, for the first time, the evidence on the prevalence and risk of violence and poor physical, mental and sexual and reproductive health outcomes amongst people who have been trafficked. If possible (i.e., if studies are not too few in number, poor quality, or too heterogeneous), a meta-analysis will be carried out to estimate the prevalence and risk of violence and key health outcomes (e.g., HIV and STI) amongst trafficked people.

This review is being conducted as part of Siân Oram’s doctoral research on the UK response to health and human trafficking. Funding from the doctoral research has been provided by the Economic and Social Research Council UK. Ethical approval for the research has been granted by the ethics committee of the London School of Hygiene & Tropical Medicine.

1. **Objectives**

The objectives of this review are to establish:

1. The prevalence of violence experienced by trafficked people whilst trafficked
2. The prevalence of physical, mental, and sexual health problems amongst trafficked people.
3. Trafficked people’s risk of experiencing violence whilst trafficked compared with that of non-trafficked people working in the same industry.
4. Trafficked people’s risk of experiencing physical, mental, and sexual health problems compared with that of non-trafficked people working in the same industry.

Analysis of the prevalence and risk of violence and of poor health consequences amongst trafficked people will be conducted separately according to gender and type of exploitation. The prevalence and risk of physical, psychological and sexual violence will be presented separately if data are available.

1. **Key Terms**
   1. **Human Trafficking**

This review uses the definition of human trafficking provided by the United Nations Optional Protocol on the Prevention, Punishment and Suppression of Trafficking in Persons, Especially Women and Children. According to this definition, trafficking is:

*“the recruitment, transportation, transfer, harbouring or receipt of persons, by means of the threat or use of force or other forms of coercion, of abduction, of fraud, of deception, of the abuse of power or of a position of vulnerability or of the giving or receiving of payments or benefits to achieve the consent of a person having control over another person, for the purpose of exploitation. Exploitation shall include, at a minimum, the exploitation of the prostitution of others or other forms of sexual exploitation, forced labour or services, slavery or practices similar to slavery, servitude or the removal of organs”*

The definition requires that a person is moved, by a variety of means, for the purpose of exploitation if they are to be considered to have been trafficked. The definition’s lack of specificity around concepts such as exploitation and control has been criticised , but its breadth is useful for the purposes of this review.

- 1. **Violence**

This review uses the WHO definition of violence, i.e., the “intentional use of physical force or power, threatened or actual, against oneself, another person, or against a group or community, that either results in or has a high likelihood of resulting in injury, death, psychological harm, mal-development, or deprivation” . The inclusion of “power” within the definition allows for the inclusion of threats, intimidation, and neglect as acts of violence .

1. **Selection Criteria**
   1. **Inclusion criteria**
      1. **Study Population**

Studies will be eligible for inclusion in the review if samples include males or females (adult or child) that self-identify or are believed by the research team to have been trafficked. If studies include trafficked people as a subset of a broader sample, data on trafficked people must be reported separately.

- - 1. **Study Setting**

No restrictions will be placed on study setting.

- - 1. **Study Characteristics**

Studies are eligible for inclusion if they present the results of published peer-reviewed research based on the following study designs: cross-sectional survey; case control study; cohort study; case series analysis; experimental study with baseline measures for the outcomes of interest; or secondary analysis of organisational records

- - 1. **Study Outcomes**

Studies are eligible for inclusion in the review if they measure the prevalence and/or the risk of physical, psychological or sexual violence whilst trafficked; and/or any reported measure of physical, mental, and sexual health problems.

No restrictions will be placed on the method of measuring physical, mental, and sexual health problems.

- 1. **Exclusion criteria**
     1. **Study Population**

Studies whose samples do not include people who self-define or are believed by the research term to have been trafficked will be excluded from the review. Studies will also be excluded from the review if they have collected data on trafficked people as a subset of a broader sample but do not report this data separately.

- - 1. **Study Characteristics**

Studies will be excluded from the review if they use a case study or qualitative design. Non-peer reviewed publications, such as theses, dissertations and reports will be excluded. General discussion papers, comments and letters, book chapters, and conference papers will be excluded.

- - 1. **Study Outcomes**

Studies which do not measure the prevalence or the risk (e.g., odds ratios, relative risk) of physical, psychological or sexual violence whilst trafficked or physical, mental, and sexual health problems amongst trafficked people, will be excluded from the review.

- 1. **Date Restrictions**

The review will examine all studies published up to the end of August 2011. No lower date restrictions will be used.

- 1. **Language Restrictions**

No language restrictions will be used.

1. **Search Strategy**
   1. **Search Terms**

The search terms that will be used in the review are as follows:

1. human trafficking.mp
2. people trafficking.mp
3. trafficking in people.mp
4. sex trafficking.mp
5. woman trafficking.mp
6. child trafficking.mp
7. trafficked people.mp
8. trafficked women.mp
9. trafficked men.mp
10. trafficked children.mp
11. forced labour.mp
12. forced labor.mp
13. forced prostitution.mp
14. sexual slavery.mp
15. 1 OR 2 OR 3 OR 4 OR 5 OR 6 OR 7 OR 8 OR 9 0R 10 OR 11 OR 12 OR 13 OR 14
16. health/
17. well-being.mp OR wellbeing.mp
18. ill-health.mp
19. illness.mp
20. “Wounds and injuries/” OR wound.mp OR injur$.mp
21. disease/
22. disability.mp
23. infection/
24. symptom.mp
25. trauma.mp
26. “mental illness”/
27. “mental disorder”/
28. anxiety/
29. depression/
30. fear/
31. guilt/
32. hostility/
33. suicide/
34. “Behavioral symptom”/
35. “Self-injurious behaviour”/
36. “Reproductive behavior” OR “Risk taking”/
37. “Sexual behavior”/
38. “Social behavior”/
39. violence/
40. rape/
41. “sexually transmitted diseases”/
42. HIV/
43. pregnancy/
44. “abortion, induced”/
45. 16 OR 16 OR 17 OR 18 OR 19 OR 20 OR 21 OR 22 OR 23 OR 24 OR 25 OR 26 OR 27 OR 28 OR 29 OR 30 OR 31 OR 32 OR 33 OR 34 OR 35 OR 36 OR 37 OR 38 OR 39 OR 40 OR 41 OR 42 OR 43 OR 44
46. protein OR membrane OR cell
47. (15 AND 45) NOT 46
    1. **Data Sources**
       1. **Electronic Databases**

The above search terms will be used to search PubMed; MEDLINE; EMBASE; Web of Science; and PsycINFO.

- - 1. **Hand Searches**

Due to resource constraints, hand searches will not be carried out.

- - 1. **Additional Sources**

Forwards and backwards citation tracking of included papers will be used to locate additional references for the review. Experts working in this area will also be asked to recommend additional studies that have not been located through the electronic literature search.

- 1. **Bias**
     1. **Publication, Location and Language Bias**

Studies that report statistically significant results are more likely to be published than those reporting no significance, to be published in a journal that is indexed in an electronic database, to be published in English, and to be cited more often . Furthermore, research conducted in developing countries or reported in a language other than English are less likely to be published in an indexed journal.

Our decision to include studies written in languages other than English may help to address publication bias. Searching a variety of electronic databases and drawing upon the supervisors of the research to identify in-press or un-indexed articles may also reduce the effects of publication and location bias.

- - 1. **Duplication Bias**

Where the literature search identifies multiple eligible papers from the same study, only the most definitive results will be included for each outcome of interest (e.g., violence, mental health).

1. **Conducting the Review**
   1. **Title and Abstract Screening**

The databases listed in section 5 will be searched using the search terms presented above. The resulting citations will be downloaded to EndNote© and duplicate citations removed. Additional citations that have been identified by forward/backwards citation tracking and the research supervisors will also be added to the EndNote© library.

The titles and abstracts of the downloaded citations will be independently evaluated by two reviewers against the inclusion and exclusion criteria for the review. If it is not possible to determine whether a paper is relevant, or if the two reviewers disagree, it will be included at this stage.

- 1. **Retrieval and Screening of Full Text Articles**

Full text copies of potentially eligible papers will be obtained. The eligibility of the full-text papers for inclusion in the review will be independently assessed by two reviewers against the inclusion and exclusion criteria. The reasons for excluding papers will be recorded (see Appendix A). If the two reviewers cannot resolve disagreements by discussion, a third reviewer will be consulted.

- 1. **Data Extraction**

Data will be extracted from by one reviewer for all included studies using a standardised extraction form. A second reviewer will independently extract data from a random sample of 20% included studies as a check. The extraction form has fields for the study bibliography, design, sample characteristics and outcomes. The outcomes of interest are experiences of violence and prevalence/risk of physical, mental, and sexual health problems amongst trafficked people. Information on sex-, age-, and industry-specific prevalence and risks will be extracted if recorded.

- 1. **Study Appraisal**

The methodological quality of studies will be appraised using an adapted version of the Critical Appraisal Skills Programme (CASP) checklist (see appendix B). Overall study quality will be assessed according to the percentage of the maximum possible quality score attained.

The quality appraisal form has 15 questions, organised into 11 domains. 2 points are available for each question, giving a maximum score of 30. A study is awarded 0 points if it does not meet the criteria or answer the question, 1 point if it partially meets the criteria or gives a partially satisfactory answer to the question, and 2 points if it fully meets the criteria or gives a fully satisfactory answer to the question. If a meta-analysis is conducted, papers which score poorly on questions relating to bias will be excluded from the analysis. Sensitivity analyses will be conducted to determine whether the exclusion of these studies from the meta-analysis makes a difference to the findings.

- 1. **Data Analysis**

Information about the study population (e.g., the country studied, the age range, sex of sample, type of exploitation, time since exploitation), sample characteristics (e.g., sample size, response rate), and method of measurement (e.g., review of medical notes, diagnostic tests, self-report), and study outcomes (e.g., prevalence and/or risk of violence and physical, mental, sexual and/or reproductive health symptoms or disorders) will be summarised.

If possible, outcome measures will be presented separately according to gender, age (adult/child) and industry of exploitation. Risk will be reported primarily using odds ratios with 95% confidence intervals. Depending on the heterogeneity of the studies, statistics will be pooled to determine the overall prevalence and risk of key health experiences and outcomes (e.g., rape, HIV). If data are pooled, the overall summary statistics will also be presented (with 95% confidence intervals).

**References**

1. Council-of-Europe. Council of Europe Convention on Action against Trafficking in Human Beings and its Explanatory Report. Warsaw 16V 2005: Council of Europe; 2005.

2. UN Convention against Transnational Organised Crime, and optional Protocol to Prevent, Suppress and Punish Trafficking in Persons, Especially Women and Children, Supplementing the United Nations Convention Against Transnational Organized Crime, (2000).

3. IOM, LSHTM, UNGIFT. Caring for Trafficked Persons: Guidance for Health Providers. Geneva: International Organization for Migration; 2009.

4. Zimmerman C, Hossain M, Yun K, Gajdadziev V, Guzun N, Tchomarova M, et al. The health of trafficked women: A survey of women entering posttrafficking services in Europe. American Journal of Public Health. 2008;98:55-9.

5. Tsutsumi A, Izutsu T, Poudyal AK, Kato S, Marui E. Mental health of female survivors of human trafficking in Nepal. Soc Sci Med. 2008 Feb 12;66:1841-7.

6. Doezema J. Who gets to choose? Coercion, consent and the UN trafficking protocol. Gender and Development. 2002 March;10(1):20-7.

7. Munro VE. Of Rights and Rhetoric: Discourses of Degradation and Exploitation in the Context of Sex Trafficking. Journal of Law and Society. 2008 June;35(2):240-64.

8. Gallagher A. Human rights and the new UN protocols on trafficking and migrant smuggling: a preliminary analysis. Human Rights Quarterly. 2001;23:975-1004.

9. WHO. WHO global consultation on violence and health: violence, a public health priority. Geneva: WHO1996.

10. Krug EL, Dahlberg L. A Global Public Health Problem World Report on Violence and Health. Geneva: WHO2002.

11. Easterbrook PJ, Gopalan R, Berlin JA, Matthews DR. Publication bias in clinical research. The Lancet. 1991;337(8746):867-72.

12. Egger M, Smith GD. Meta-analysis bias in location and selection of studies. BMJ. 1998;316:61.

**Appendix A:**

**Checklist for Screening Full Text Papers**

Does the paper meet **each** of the following inclusion criteria?

| **Inclusion criteria** | **If yes tick box** |
| --- | --- |
| Study is published in a peer-reviewed journal |  |
| Study uses an eligible study design (experimental study with baseline measure, cohort study, case-control study, cross-sectional study, secondary analysis of organisational records, case series) |  |
| Sample includes participants who report or are believed to have been trafficked |  |
| Study results include the prevalence or risk of violence and/or physical, mental, and/or sexual or reproductive health symptoms or disorders. |  |

If the paper does not meet **all** of the above criteria, please indicate below the reasons why:

| **Exclusion criteria** | **If yes tick box** |
| --- | --- |
| Study is published in a book, report conference paper, dissertation/thesis, general comment paper, letter, editorial or other non-peer reviewed format. |  |
| Study uses an ineligible study design (single case study, qualitative interview, focus group interviews) |  |
| Sample does not include participants who have been trafficked (or includes who have been trafficked but does not provide appropriately disaggregated data) |  |
| Study does not measure violence and/or physical, mental, and/or sexual or reproductive health symptoms or disorders. |  |

If the paper meets any of the exclusion criteria do not proceed with data extraction.

**Appendix B:**

**Quality Appraisal Form**

Questions should be scored as follows:

0 – study does not meet criteria/answer question

1 – study partially meets criteria/gives a partially satisfactory answer to the question

2 – study fully meets criteria/gives a fully satisfactory answer to the question

n/a - question is not applicable

| Question | Considerations | Comments | Score |
| --- | --- | --- | --- |
| 1 – Does the study address a clearly focused question? | -focused in terms of population of interest  -focused in terms of outcomes studied |  |  |
| 2 – Is the study design appropriate to address the research question? |  |  |  |
| 3.1 – Does the study uses an appropriate sampling method? | -sampling method  -time frame  -sample size |  |  |
| 3.2 – Is the study sample appropriate to address the research question? | -sample characteristics clearly described  -clear inclusion and exclusion criteria  - appropriate controls  -representativeness of sample |  |  |
| 3.3 – Is the level of non-participation tolerable? | -level of non-participation  - comparison of non-participants and participants  - impact of non-participation |  |  |
| 4 – Is the exposure (trafficking) appropriately assessed? | -definition of trafficking is provided  -suitability of the indicators used  -potential for bias |  |  |
| 5 – Are the outcomes (violence and/or health symptoms and disorders) appropriately assessed? | -validated clinical and/or survey instruments used to assess outcomes |  |  |
| 6 – Are known confounders accounted for? | -key confounders identified  -design and analysis addresses confounders |  |  |
| 7.1 – Are appropriate statistical analyses conducted? |  |  |  |
| 7.2 – Are prevalence/risk measures reported with confidence intervals? |  |  |  |
| 7.3 – How precise are the results? |  |  |  |
| 8 - Were ethical issues appropriately considered? | -informed consent  -safeguarding anonymity, confidentiality and safety  -availability of support and referral options  -fieldworker training |  |  |
| 9 - Do the findings support the conclusions? |  |  |  |
| 10 - Are the findings generalisable? |  |  |  |
| 11 - Study results fit with existing evidence |  |  |  |

**Total Score (from a maximum of 30): …………………..**
