# Supplementary material for: Prevalence and Risk of Violence and the Physical, Mental, and Sexual Health Problems Associated with Human Trafficking: Systematic Review
Source: PLoS Med. 2012 May 29;9(5):e1001224. doi: 10.1371/journal.pmed.1001224 (PMC3362635; doi:10.1371/journal.pmed.1001224)
Supplement: Text S3 — Search terms used for Ovid Medline, EMBASE, and PSYCInfo. (DOC) [file pmed.1001224.s003.doc]

Text S3

Search terms used for Ovid Medline, EMBASE and PSYCInfo

1. human trafficking.mp OR people trafficking.mp OR trafficking in people.mp OR sex trafficking.mp OR woman trafficking.mp OR child trafficking.mp OR trafficked people.mp OR trafficked women.mp OR trafficked men.mp OR trafficked children.mp OR forced labour.mp OR forced labor.mp OR forced prostitution.mp OR sexual slavery.mp
2. health/ OR well-being.mp OR wellbeing.mp OR ill-health.mp OR illness.mp OR “Wounds and injuries/” OR wound.mp OR injur$.mp OR disease/ OR disability.mp OR infection/ OR symptom.mp OR trauma.mp OR “mental illness”/ OR “mental disorder”/ OR anxiety/ OR depression/ OR fear/ OR guilt/ OR hostility/ OR suicide/ OR “Behavioral symptom”/ OR “Self-injurious behaviour”/ OR “Reproductive behavior” OR “Risk taking”/ OR “Sexual behavior”/ OR “Social behavior”/ OR violence/ OR rape/ OR “sexually transmitted diseases”/ OR HIV/ OR pregnancy/ OR “abortion, induced”/)
3. (protein OR membrane OR cell)
4. (1 AND 2) NOT 3
