# Supplementary material for: Prevalence and Risk of Violence and the Physical, Mental, and Sexual Health Problems Associated with Human Trafficking: Systematic Review
Source: PLoS Med. 2012 May 29;9(5):e1001224. doi: 10.1371/journal.pmed.1001224 (PMC3362635; doi:10.1371/journal.pmed.1001224)
Supplement: Text S4 — Quality appraisal checklist. (DOC) [file pmed.1001224.s004.doc]

Text S4

Quality Appraisal Checklist

Scoring

0 – Study does not meet criteria/answer question

1 – Study partially meets criteria/gives a partially satisfactory answer to the question

2 – Study fully meets criteria/gives a fully satisfactory answer to the question

| Question | Considerations | Comments | Score |
| --- | --- | --- | --- |
| 1 – Does the study address a clearly focused question? | -focused in terms of population of interest  -focused in terms of outcomes studied |  |  |
| 2 – Is the study design appropriate to address the research question? |  |  |  |
| 3 – Does the study use an appropriate sampling method? | -sampling method  -time frame  -sample size |  |  |
| 4 – Is the study sample appropriate to address the research question? | -sample characteristics clearly described  -clear inclusion and exclusion criteria  - appropriate controls  -representativeness of sample |  |  |
| 5 – Is the level of non-participation tolerable? | -level of non-participation  - comparison of non-participants and participants  - impact of non-participation |  |  |
| 6 – Is the exposure (trafficking) appropriately assessed? | -definition of trafficking is provided  -suitability of the indicators used  -potential for bias |  |  |
| 7 – Are the outcomes (violence and/or health symptoms and disorders) appropriately assessed? | -validated clinical and/or survey instruments used to assess outcomes |  |  |
| 8 – Are known confounders accounted for? | -key confounders identified  -design and analysis addresses confounders |  |  |
| 9 – Are appropriate statistical analyses conducted? |  |  |  |
| 10– Are prevalence/risk measures reported with confidence intervals? |  |  |  |
| 11– How precise are the results? |  |  |  |
| 12 - Were ethical issues appropriately considered? | -informed consent  -safeguarding anonymity, confidentiality and safety  -availability of support and referral options  -fieldworker training |  |  |
| 13 - Do the findings support the conclusions? |  |  |  |
| 14 - Are the findings generalisable? |  |  |  |
| 15 - Study results fit with existing evidence |  |  |  |

Total score (/30): add scores for all questions.

Selection quality score: (/6): add scores for questions 3, 4 and 5

Measurement quality score (/6): add scores for questions 6, 7 and 8.
